# Japan's COVID-19 response: underutilized beds and misused funds

**DOI:** 10.3389/fpubh.2023.1277746

**Published:** 2023-10-02

**Authors:** Yoshika Saito, Akihiko Ozaki, Tetsuya Tanimoto, Morihito Takita, Masahiro Kami

**Affiliations:** ^1^Medical Governance Research Institute, Tokyo, Japan; ^2^Faculty of Medicine, Kyoto University, Kyoto, Japan; ^3^Department of Breast and Thyroid Surgery, Jyoban Hospital of Tokiwa Foundation, Iwaki, Japan; ^4^Navitas Clinic Tachikawa, Department of Internal Medicine, Tachikawa, Japan

**Keywords:** COVID-19, health economics, Japan, public health, resource allocation

Considering that infection control measures serve as a paramount example of public health policy, it is essential that each national government takes a leading role in their implementation. However, the reality of this implementation is undeniably influenced by the available human and medical resources, as well as the unique cultural and historical contexts of each country. Clearly, executing this implementation process is far from straightforward due to the multitude of stakeholders involved and the numerous challenges to overcome. Nonetheless, critical evaluations of such processes have not been conducted adequately to date.

In this regard, the recent COVID-19 pandemic has offered a distinct opportunity to examine these mechanisms. This pandemic posed an unprecedented challenge to global healthcare systems, compelling countries to mobilize substantial resources and quickly adapt their strategies to curb the spread of the virus. Japan is no exception. As a country neighboring China, the origin of SARS-CoV-2, Japan has significantly felt the impact of the pandemic. As of June 25, 2023, Japan has reported 3,803,572 COVID-19 cases and 74,694 fatalities since the onset of the pandemic (1). With this in mind, we aim to highlight and scrutinize Japan's experience, in hopes of providing essential insights for the effective management of future disasters and crises.

Japan, along with many other countries, has made substantial fiscal investments in its pandemic response. In fact, the Japanese government allocated $550 billion in the 2020 fiscal year to COVID-19 initiatives (2), a staggering amount that surpasses the $270 billion expended over a decade on recovery efforts following the 2011 Great East Japan Earthquake (3). Furthermore, public expenditures related to COVID-19 in Japan account for 45% of its GDP. This figure ranks second among advanced countries, following Italy, but surpasses Italy in terms of the actual amount spent (4). Naturally, various policies of the country were questionable due to misalignment with the actual circumstances. A prime example of this discrepancy was the policy related to the allocation of hospital beds for COVID-19 patients.

In April 2020, the Japanese government began subsidizing up to $530 per day for each general hospital bed and $3,125 per day for each ICU bed, in an effort to secure sufficient hospital space for COVID-19 patients (5). However, by August 2021 and February 2022, the utilization rate of these beds had fallen below 50% in 28.5% (136/476) and 27.5% (136/493) of facilities, respectively (5). Nonetheless, a substantial number of patients in the metropolitan area of Tokyo encountered significant difficulties in securing transfers to medical institutions via emergency medical services. This challenge was faced by patients irrespective of whether their illnesses were related to COVID-19, and notably, it occurred despite the consistent availability of hospital beds (6). This unfortunate situation resulted in fatalities among both COVID-19 and non-COVID-19 patients (6). The significant underutilization of beds, coupled with a limited capacity to accept emergency cases in these hospitals, may not be attributed solely to inadequate efforts by individual institutions. Instead, it points to potential inherent flaws in the policies themselves.

It is worth examining the Japan Community Health care Organization (JCHO) as a notable example of the potential pitfalls of these policies. The JCHO was established as an independent administrative institution to address public health crises and operates 57 public hospitals that maintain regional healthcare and promoting public health. According to the act on JCHO, these hospitals must address significant public health risks and provide medical care for infectious disease patients (7). Given that COVID-19 qualifies as such an emergency, the JCHO was compelled to undertake measures like reserving specific beds for COVID-19 patients. However, as of the end of July 2021, these reserved beds constituted only about 5% of the total hospital beds (8). As a result, in October 2021, the Ministry of Health, Labor and Welfare formally requested an increase in the number of reserved beds (9).

There are at least two reasons behind this situation. First, a lack of preparedness. The JCHO failed to fully embrace its role and did not establish an effective system for admitting COVID-19 patients, including guaranteeing adequate staffing. For instance, in the fiscal year 2020, The subsidies received by JCHO increased by $214 million compared to the fiscal year 2019 (10, 11). Yet, this sum was not allocated toward enhancing the medical infrastructure. For example, labor costs only increased by ~$18 million compared to the previous year (10, 11), suggesting it was likely used for other purposes. It is possible that JCHO had a strong inclination to prioritize maintaining good financial management rather than actively fulfilling its role during the crisis.

Secondly, policy inconsistencies contributed to the situation. In December 2019, the government issued a notification stating that COVID-19 should be managed by designated core medical institutions equipped with special resources to handle emerging infectious diseases, allowing non-designated hospitals to refuse treatment (12). Given the role of JCHO, all 57 of its hospitals should have been designated institutions, yet only 13 hospitals received such a designation (13). Consequently, the remaining 44 hospitals had the option to decline COVID-19 related care. There are no penalties for not received COVID-19 patients, and from a business perspective, it may be advantageous to refuse such cases. Thus, the policy's application in controlling public hospitals can be deemed a failure in Japan.

Furthermore, we could contemplate a more systemic reason behind this incident, which involves the allocation of human and medical resources among private and public medical institutions. While Japan embraces universal health coverage, unlike the United Kingdom with the NHS, the presence of public hospitals is comparatively small. Japan's public hospitals account for only 27% of total beds, which is significantly lower than the 100% in the United Kingdom (estimated) and 61% in France (as of 2019) (14). Furthermore, medical schools and their affiliated hospitals operate independently from government control although a significant portion of their medical fees are reimbursed through the national health insurance system. Hence, when faced with the demands of the pandemic, private hospitals grappled with the challenge of cultivating an adequate number of sufficiently trained experts within their institutions capable of responding to such crises. Consequently, it was not practical to anticipate that public hospitals in Japan would bear the majority of COVID-19 treatment responsibilities. In fact, the Japanese government gradually transitioned toward a policy of using private hospitals as the primary care centers for the COVID-19 pandemic, which proved to be effective.

In this context, it becomes apparent that the policies advocated by some, which aimed to empower the government to secure more beds for COVID-19 patients, may not have been as effective as anticipated. It is noteworthy that these policies did not yield successful outcomes even in China, where the government has essentially control over all hospital beds. A sudden relaxation of regulations resulted in a nearly 9-fold surge in the total number of infections within a mere 2 months (15). This surge resulted in a strain on medical resources due to the lack of adequate hospital bed provision beforehand. Instead, Japan should focus on fostering collaboration among various public and private hospitals and local governments within each community. While hospitals in Japan are currently organized in a fragmented manner based on their respective founding bodies, efforts should be made to enhance horizontal collaboration at the regional level to better prepare for future emergencies.

Indeed, some successful cases have been observed within the country. In Fukushima Prefecture, a healthcare management system known as the Fukushima Model has been implemented. This model represents a collaboration among hospitals, the local university, and local governments, and it evolved from the patient evacuation procedures established during the Great East Japan Earthquake. Under this system, COVID-19 patients are classified according to severity, and after coordination by the local medical school, they receive treatment at designated hospitals. Within this network, medical information is shared, enabling comprehensive patient management and facilitating smooth transfers between hospitals (16). In fact, the percentage of infected people in Fukushima Prefecture is lower than the national average in Japan (17). Thus, it is quite enlightening to observe that hospitals which proactively sought and sustained collaboration with local communities and other healthcare institutions, leveraging bottom-up approaches, proved to be effective during the COVID-19 pandemic in the Japanese society.

Across Japan, the number of secured beds has consistently risen since April 2021, following the mandate for hospitals to allocate dedicated beds for COVID-19 patients (18). However, these measures have proven to be inadequate. The waiting list for hospitalizations has expanded in tandem with the surge in COVID-19 cases, peaking at ~1,500 in August 2021 and reaching around 4,000 by February 2022 (18).

Significantly, we believe that this argument is relevant to various countries with similar distributions of medical and human resources. A case in point is the United States. Generally, the health systems of the United States and Japan are considered distinct, yet they both have a strong presence of private sectors in their medical communities. Indeed, in the United States, areas that have fostered more partnerships between local public institutions, healthcare facilities, and hospitals have experienced a 9–10% decrease in the risk of higher case-fatality rates (19). Consequently, these collaborations allowed for the maintenance of medical resources, efficient distribution, and more effective differentiation in hospital functions, enabling flexible responses to increasing patient numbers.

In conclusion, we illuminated Japan's experiences during the COVID-19 pandemic, with a particular emphasis on health policy concerning hospital bed allocation between the private and public sectors. In countries like Japan, where the private sector plays a significant role in the medical community, we posit that a policy fostering harmonious collaboration between private and public hospitals could operate effectively, even during a pandemic that requires strong governmental leadership. Policymakers, healthcare professionals, and administrative staff must bear in mind that a successful response requires an approach that is attuned to each country's unique culture and health system, as well as the scale and nature of the crises at hand. This point, highlighted by Japan's experience during the COVID-19 pandemic, should be considered and applied when managing crises that may arise in different regions or times.

## Author contributions

YS: Writing—original draft, Writing—review and editing, Conceptualization. AO: Supervision, Writing—review and editing, Conceptualization. TT: Writing—review and editing, Conceptualization. MT: Writing—review and editing, Conceptualization. MK: Supervision, Writing—review and editing, Conceptualization.
